# New species of *Dermoergasilus* Ho & Do, 1982 (Copepoda: Cyclopoida: Ergasilidae) parasitizing endemic cichlid *Paretroplus polyactis* (Bleeker) in Madagascar

**DOI:** 10.1017/S0031182024000088

**Published:** 2024-03

**Authors:** Robert Míč, Eva Řehulková, Andrea Šimková, Jeanne Rasamy Razanabolana, Mária Seifertová

**Affiliations:** 1 Department of Botany and Zoology, Faculty of Science, Masaryk University, Kotlářská 2, 611 37, Brno, Czech Republic; 2 Department of Animal Biology, Faculty of Science, University of Antananarivo, BP 906 Antananarivo 101, Madagascar

**Keywords:** Cichlids, COI, *Dermoergasilus*, diversity, Ergasilidae, Madagascar, parasitic crustaceans, phylogeny, rDNA

## Abstract

*Dermoergasilus madagascarensis* n. sp. is described from the gills of *Paretroplus polyactis*, an endemic cichlid fish in Madagascar, using a combined morphological (light microscopy and SEM) and molecular approach (partial 18S rDNA, 28S rDNA, and COI sequences). The new species is characterized mainly by possessing: (i) roughly pentagonal cephalosome; (ii) antennal endopodal segments covered with slightly inflated membrane; (iii) maxillule bearing 2 equally long outer setae and a minute inner seta; (iv) interpodal sternites of swimming legs ornamented with 3–4 rows of spinules; (v) genital segment and first abdominal somite both barrel-shaped; and (vi) a caudal ramus projecting into a digitiform process with inconspicuous terminal seta and bearing 3 terminal setae. The obtained DNA sequences of Malagasy species represent the first molecular data for species of *Dermoergasilus*. The 28S rDNA phylogeny showed the affiliation of *D. madagascarensis* n. sp. to Ergasilidae and its sister relationship with cosmopolitan *Ergasilus sieboldi* von Nordmann, 1832. The first checklist for all species of *Dermoergasilus* is provided.

## Graphical abstract

**Figure:**
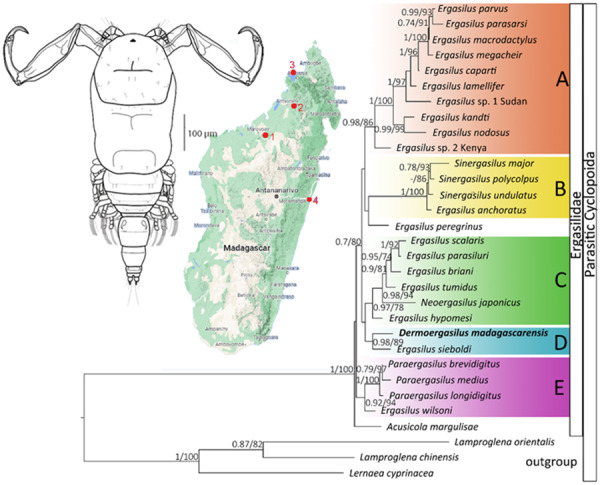

## Introduction

*Dermoergasilus* Ho and Do, 1982 currently includes 12 valid species parasitizing freshwater, marine and brackish water fishes in Indian, Indo-West Pacific, Palaearctic and Afrotropic regions (Dogiel and Akhmerov, 1952; Cressey and Collette, 1970; Ho and Do, 1982; Byrnes, 1986; Oldewage and Van As, 1988; Ho *et al*., 1992; Kabata, 1992; El-Rashidy and Boxshall, 1999; El-Rashidy and Boxshall, 2001; Hassan *et al*., 2009; Ali and Adday, 2019). The host spectrum of *Dermoergasilus* species is broad and comprises various fishes belonging to 14 families, including mostly Mugilidae (12 species), Belonidae (4 species) and Sparidae (3 species). The number of host species parasitized by a *Dermoergasilus* species ranges from 1 (*Dermoergasilus cichlidus* Ali and Adday, 2019, *Dermoergasilus curtus* El-Rashidy and Boxshall, 2001 and *Dermoergasilus semicoleus* Cressey and Collette, 1970) to 8 [*Dermoergasilus amplectens*(Dogiel and Akhmerov, 1952)] (Table 1).

**Table 1.** Checklist of *Dermoergasilus* including host species, locality and site of collection

Dermoergasilus species	Host species	Host family	Locality	Site	References
*D. acanthopagri* Byrnes, 1986	*Acanthopagrus australis*	Sparidae	Gladstone, Australia	Gills	Byrnes (1986)
	*Acanthopagrus berda*	Sparidae	Daintree, Australia	Gills	Byrnes (1986)
	*Acanthopagrus butcheri*	Sparidae	Perth, Eden, Australia	Gills	Byrnes (1986)
*D. amplectens* (Dogiel and Akhmerov, 1952)	*Chanos chanos*	Chanidae	Poonthura, Trivandrum, India	Gills	Ho *et al*. (1992)
	*Valamugil seheli* (=*Crenimugil sehel*)	Mugilidae	Veli Lake, Trivandrum, India	Gills	Ho *et al*. (1992)
	*Gerres setifer*	Gerreidae	Neendakara, India	Gills	Ho *et al*. (1992)
	*Liza argentea* (=*Gracilimugil argenteus*)	Mugilidae	Serpentine Creek, Brisbane, Australia	Unknown	Kabata (1992)
	*Hyporhamphus xanthopterus*	Hemiramphidae	Poonthura, Trivandrum, India	Gills	Ho *et al*. (1992)
	*Megalops cyprinoides*	Megalopidae	Killiyar River, Trivandrum, India	Gills	Ho *et al*. (1992)
	*Mugil cephalus*	Mugilidae	Tumen-Ula River, Russia	Unknown	Dogiel and Akhmerov (1952)
			Kojima Bay, Okayama Prefecture, Japan	Gills	Ho and Do (1982)
			Tallebudgera Creek, South Queensland, Australia	Unknown	Kabata (1992)
			Mackay Fish Board, Australia	Unknown	Kabata (1992)
			Wakanoura, Japan	Gills	El-Rashidy and Boxshall (2001)
			Tsushima, Japan	Gills	El-Rashidy and Boxshall (2001)
			Kowie River, South Africa	Gills	El-Rashidy and Boxshall (2001)
	*Etroplus maculatus* (=*Pseudetroplus maculatus*)	Cichlidae	Veli Lake, Trivandrum, India	Gills	Ho *et al*. (1992)
*D. cichlidus* Ali and Adday, 2019	*Coptodon zillii*	Cichlidae	Shatt Al-Arab River, Al-Hartha District, Iraq	Gills	Ali and Adday (2019)
			Pond of Marine Sciences Centre, Basrah, Iraq	Gills	Ali and Adday (2019)
*D. coleus* (Cressey in Cressey and Collette, 1970)	*Strongylura urvillii*	Belonidae	Philippines	Gills	Cressey and Collette (1970)
	*Strongylura strongylura*	Belonidae	Cagayan de Misamis, Mindanao, Philippines	Gills	Cressey and Collette (1970)
			Sandakan Bay, Borneo, Malaysia	Gills	Cressey and Collette (1970)
			Porto Novo, Madras, India	Gills	Cressey and Collette (1970)
	*Xenentodon cancila*	Belonidae	Travancore, India	Gills	Cressey and Collette (1970)
			Calcutta, India	Gills	Cressey and Collette (1970)
*D. curtus* El-Rashidy and Boxshall, 2001	*Rhinomugil squamipinnis* (=*Rhinomugil corsula*)	Mugilidae	Alahabad, India	Gills	El-Rashidy and Boxshall (2001)
*D. intermedius* (Kabata, 1992)	*Maccullochella macquariensis*	Percichthyidae	Moreton Bay, Queensland, Australia	Unknown	Kabata (1992)
	*Tandanus tandanus*	Plotosidae	Macintyre River, Queensland, Australia	Unknown	Kabata (1992)
			Taroon, Queensland, Australia	Unknown	Kabata (1992)
	*Fluvialosa richardsoni* (=*Nematalosa erebi*)	Dorosomatidae	Macintyre River, Queensland, Australia	Unknown	Kabata (1992)
	*Plectroplites ambiguus* (=*Macquaria ambigua*)	Percichthyidae	Macintyre River, Queensland, Australia	Unknown	Kabata (1992)
*D. longiabdominalis* El-Rashidy and Boxshall, 2001	*Valamugil engeli* (=*Osteomugil engeli*)	Mugilidae	Calabato, Mindanao, Philippines	Gills	El-Rashidy and Boxshall (2001)
	*Valamugil cunnesius* (=*Osteomugil cunnesius*)	Mugilidae	Tamatave, Madagascar	Gills	El-Rashidy and Boxshall (2001)
			Mindanao, Philippines	Gills	El-Rashidy and Boxshall (2001)
			Mangalore, India	Gills	El-Rashidy and Boxshall (2001)
***D. madagascarensis* n. sp.**	***Paretroplus polyactis***	**Cichlidae**	**Canal des Pangalanes (at Andevoranto), Madagascar**	**Gills**	**Present study**
*D. mugilis* Oldewage and Van As 1988	*Mugil cephalus*	Mugilidae	Mouth of Keurbooms River, Cape Province, South Africa	Gills	Oldewage and Van As (1988)
			Bushman's River mouth, South Africa	Gills	Oldewage and Van As (1988)
*D. occidentalis* Hassan *et al.*, 2009	*Tandanus bostocki*	Plotosidae	Jalbarragup, Blackwood River, Western Australia	Gills	Hassan *et al*. (2009)
	*Galaxias occidentalis*	Galaxiidae	Swan River, Western Australia	Gills	Hassan *et al*. (2009)
*D. semiamplectens* El-Rashidy and Boxshall, 2001	*Sicamugil hamiltoni*	Mugilidae	Sittang River, Myanmar	Gills	El-Rashidy and Boxshall (2001)
	*Valamugil cunnesius* (=*Osteomugil cunnesius*)	Mugilidae	China	Gills	El-Rashidy and Boxshall (2001)
	*Liza subviridis* (=*Planiliza subviridis*)	Mugilidae	Calcutta, India	Gills	El-Rashidy and Boxshall (2001)
	*Liza parsia* (=*Planiliza parsia*)	Mugilidae	Calcutta, India	Gills	El-Rashidy and Boxshall (2001)
*D. semicoleus* (Cressey in Cressey and Collette, 1970)	*Strongylura krefftii*	Belonidae	Oenpalli, Alligator River, Australia	Gills	Cressey and Collette (1970)
*D. varicoleus* Ho *et al*., 1996	*Liza abu* (=*Planiliza ab*)	Mugilidae	Shatt Al-Arab River, Iraq		Khamees and Mhaisen (1995) and Ho *et al*. (1996)
	*Liza subviridis* (=*Planiliza subviridis*)	Mugilidae	Calcutta, India	Gills	El-Rashidy and Boxshall (2001)
			Orissa, India	Gills	El-Rashidy and Boxshall (2001)
			Madras, India	Gills	El-Rashidy and Boxshall (2001)
			Bombay, India	Gills	El-Rashidy and Boxshall (2001)
	*Planiliza tade*	Mugilidae	Veli Lake, Trivandrum, India	Gills	Ho *et al*. (1992)
	N/A	Cyprinidae		Unknown	Ali and Adday (2019)
	N/A	Siluridae		Unknown	Ali and Adday (2019)
*Dermoergasilus* sp.	*Cyprinus carpio*	Cyprinidae	Marine Sciences Centre ponds, Basrah, Iraq	Gills	Ahmed and Ali (2013)

N/A, data not available.

The valid names of fish hosts are given in parentheses.

*Dermoergasilus* was proposed by Ho and Do (1982) to include 3 previously described species of *Ergasilus* (i.e. *Ergasilus amplectens* Dogiel and Akhmerov, 1952; *Ergasilus coleus* Cressey and Collette, 1970; *Ergasilus semicoleus* Cressey and Collette, 1970) possessing a combination of the following characters: (i) antenna, except terminal claw, covered with inflated transparent membrane; (ii) paired caudal rami each with a digitiform process; and (iii) middle segment of endopod of legs II and III possessing a single seta. Later, Byrnes (1986) described *Dermoergasilus acanthopagri* Byrnes, 1986 from sea breams (Sparidae) in Australia. Nevertheless, Gussev (1987) questioned the validity of the genus when he found several *Ergasilus* species possessing the antennal transparent membrane. Meanwhile, Oldewage and Van As (1988) described *Dermoergasilus mugilis* Oldewage and Van As, 1988 from grey mullet (Mugilidae) in Africa. Kabata (1992) confirmed the validity of the genus and stated that even just the digitiform process on paired caudal rami distinguishes *Dermoergasilus* from *Ergasilus*. The importance of the transparent membrane on antenna is also questioned since it is not well developed at some species of *Dermoergasilus,* and on the contrary, there are some species of *Ergasilus* which have transparent inflated membrane around the antenna (e.g. *E. acusicestraeus* El-Rashidy and Boxshall, 1999). Kabata (1992) described *Ergasilus intermedius* Kabata, 1992 and stated that this species is an intermediate form between *Ergasilus* and *Dermoergasilus*, later El-Rashidy and Boxshall (1999) transferred this species to *Dermoergasilus*. *Dermoergasilus varicoleus* Ho *et al*. (1992) parasitizing *Planiliza tade* (Fabricius) was described in India (Ho *et al*., 1992), whereas El-Rashidy and Boxshall (2001) described 3 species of *Dermoergasilus* from 6 species of grey mullet hosts (see Table 1): *D. longiabdominalis* El-Rashidy and Boxshall, 2001; *D. semiamplectens* El-Rashidy and Boxshall, 2001; and *D. curtus* El-Rashidy and Boxshall, 2001. *Dermoergasilus occidentalis* Hassan *et al.*, 2009 was described from eeltail catfishes (Plotosidae) and galaxiids (Galaxiidae) in Australia (Hassan *et al*., 2009). Ahmed and Ali (2013) reported *Dermoergasilus* sp. from common carp (*Cyprinus carpio* L.) in Iraq but did not provide further morphological identification. Most recently, *Dermoergasilus cichlidus* Ali and Adday, 2019 was described from redbelly tilapia [*Coptodon zillii* (Gervais)] in Iraq (Ali and Adday, 2019).

Until now, there are only a few parasitic crustacean records from freshwater fishes in Madagascar. Fryer (1968) questioned whether it is due to the lack of scientific interest or because of their true absence. The only record of a parasitic copepod on this island is *Dermoergasilus longiabdominalis* El-Rashidy and Boxshall, 2001 from *Osteomugil engeli* (Bleeker) (El-Rashidy and Boxshall, 2001). From other parasitic crustaceans recorded in the region only the occurrence of parasitic isopod *Cymothoa borbonica* Schioedte & Meinert, 1884 from the mouth of the freshwater cichlid fish *Ptychochromis oligacanthus* (Bleeker) is reported by (Trilles, 1975).

The other parasitic crustaceans recorded from this area are associated with the marine fish species (e.g. Barnard, 1960; Cressey, 1963; Trilles, 1975, 1979, 2008; Benz, 2006); or mud shrimps (Humes *et al*., 1958); sea stars (Humes and Cressey, 1958; Humes and Ho, 1966; Humes, 1971); gorgonaceans (Humes, 1974); holothurians (Humes and Cressey, 1959, 1961; Humes, 1967); corals (Humes, 1962; Humes and Frost, 1964; Humes and Ho, 1967); molluscs (Humes and Ho, 1965); antipatharians (Humes, 1967).

During the investigation of gill parasites of cichlid fishes in Madagascar, *Dermoergasilus* specimens were collected from the gills of *Paretroplus polyactis*. Description of new *Dermoergasilus* species was performed using morphological study (light and SEM microscopy), and a molecular study using ribosomal and mitochondrial DNA sequences (partial 18S rDNA, 28S rDNA and COI sequences). In addition, to investigate the relationship of *D. madagascarensis* n. sp. to other representatives of Ergasilidae, phylogenetic analyses were performed.

## Materials and methods

### Fish collection

**Figure 1.**
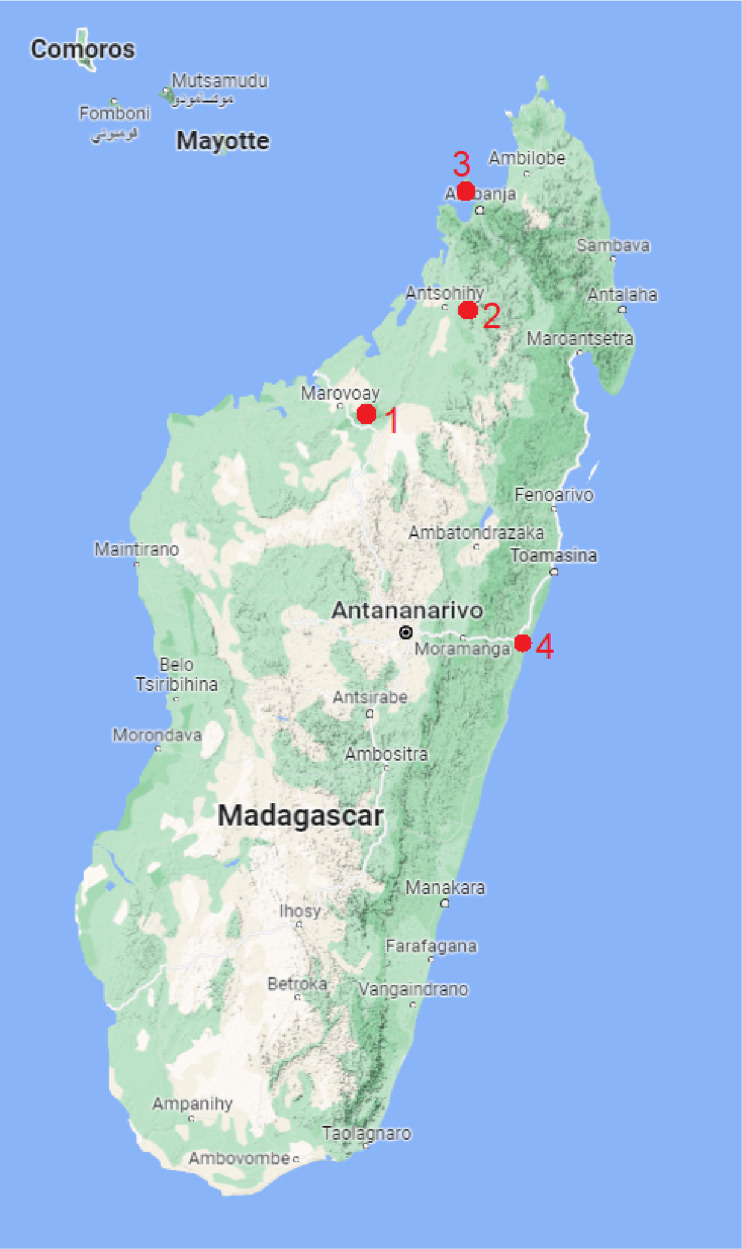
Map of Madagascar indicating the sampling localities: (1) Lake Ravelobe; (2) Anjingo River; (3) crater lakes of Mont Passot; (4) Canal des Pangalanes.

During a parasitological survey in April 2016, 100 fish specimens were examined for the presence of metazoan parasites (see Supplementary Table 1) Examined fish included mainly representatives of the family Cichlidae (92 specimens), some non-cichlid fishes living in sympatry with cichlids were also examined [4 specimens of Gobiidae (*Glossogobius giuris* (Hamilton) and *Glossogobius* sp.)], 2 specimens of Mugilidae [*Osteomugil robustus* (Günther) and *Planiliza macrolepis* (Smith) and Aplocheilidae (*Pachypanchax omalonotus* (Duméreil))]. Fishes were sampled in 4 localities (Fig. 1): (1) Lake Ravelobe (Ankarafantsika National Park) 16°18′23.14″S–46°48′43.32″E, (2) the Anjingo River (near Antsohihy) 14°50′40.89″S–48°14′43.36″E, (3) the crater lakes of Mont Passot (on Nosy Be Island) 13°19′1.84″S–48°14′3.60″E, and (4) the Canal des Pangalanes (at Andevoranto) 18°57′17.50″S–49°6′29.90″E. These areas belong to the eastern basins and freshwater systems of north-western Madagascar, all recognized as hotspots of Malagasy fish diversity (Benstead *et al*., 2003). All fish specimens were transported alive to the field laboratory, sacrificed by severing the spinal cord, and dissected within 48 h following classical parasitological dissection procedure (Ergens and Lom, 1970). Fish specimens were measured and identified by local co-workers familiar with the fish fauna, and the identification was subsequently confirmed using sequences of the cytochrome mitochondrial gene (see Šimková *et al.*, 2019 for detailed information). The present study was part of a larger investigation concerning transmission of parasites from introduced cichlids to native Malagasy fish (Šimková *et al.*, 2019).

### Parasite collection and identification

Live copepods were collected from the gills using fine needles and processed for morphological and molecular purposes, as described in Míč *et al*. (2023). The mounted specimens in GAP (mixture of glycerine and ammonium picrate) or pure glycerine were studied using an Olympus BX61 microscope equipped with phase contrast optics. Drawings of the copepods were made using an Olympus drawing attachment and edited with a graphic tablet (Wacom Intuos5 Touch) compatible with Adobe Illustrator and Adobe Photoshop (Adobe Systems Inc., San Jose, CA, USA). All measurements (in micrometers) were taken using digital image analysis software (Olympus Stream Motion v. 1.9.3) and are presented as the range followed by the mean (*n* = 10).

For scanning electron microscope analysis, 5 specimens fixed in 70% ethanol were dehydrated in an increasing ethanol grades, dried in a CPD 030 critical point drying apparatus (Bal-tec, Balzers, Liechtenstein) using liquid CO_2_, mounted on aluminium stubs with double sided adhesive discs, coated with gold in a SCD 040 sputter coating unit (OC Oerlikon Balzers Coating, Balzers, Liechtenstein) and examined in a VEGA scanning electron microscope operating at 20 kV.

For comparative purposes, specimens of the following 4 previously described species of *Dermoergasilus* available in the Natural History Museum (London, UK; BMNH) were examined: *D. amplectens* (BMNH 1999.1399-1401), *D. longiabdominalis* (BMNH 1999.1321), *D. semiamplectens* (BMNH 1999.1341-1374; BMNH 1999.1376-1377) and *D. varicoleus* (BMNH 1999.1412-1417).

The type specimens of the copepods collected in the present study were deposited in the Institute of Parasitology, Czech Academy of Sciences, České Budějovice, Czech Republic. Prevalence (percentage of infected fish) and mean intensity of infection (mean number of parasites per infected host) were calculated following Bush *et al*. (1997).

### Molecular and phylogenetic analyses

Genomic DNA was isolated separately from each parasite specimen (or a part of its body) using DNeasy®Blood & Tissue Kit (Qiagen, Hilden, Germany) according to the manufacturer's instructions. For molecular characterization, partial sequences of 1 mitochondrial gene (COI) and 2 nuclear ribosomal regions (18S and 28S rDNA) were amplified by using the primer sets listed in Table 2. PCRs for 18S and 28S rDNA were carried out in a total volume of 20 *μ*L containing 3 *μ*L of DNA extract, 1× PCR buffer (Fermentas), 2.5 mm MgCl_2_, 0.2 mm of each dNTP, 0.2 *μ*m of each primer, 0.1 BSA and 1 U of Taq polymerase (Fermentas). Amplification was performed under the following conditions: 94°C for 5 min; 39 cycles of 94°C for 30 s; an annealing temperature of 52°C for 30 s; and 72°C for 1 min, with a final extension step at 72°C for 5 min. PCR for COI was carried out in a total volume of 50 *μ*L containing 1 *μ*L of DNA extract, 1× PCR buffer (Fermentas), 2.5 mm MgCl_2_, 0.5 mm of each dNTP, 0.5 *μ*m of each primer, 0.1 BSA and 2 U of Taq polymerase (Fermentas). Amplification was performed under the following conditions: 95°C for 5 min; 40 cycles of 95°C for 1 min; an annealing temperature of 45°C for 1 min; and 72°C for 30 s, with a final extension step at 72°C for 7 min. The PCR amplicons were checked by electrophoresis on 1.5% agarose gels stained with Good View™ (Amplia s.r.o., Bratislava, Slovakia), and PCR products of the required length were purified using ExoSAP-IT™ (Affymetrix Inc., Santa Clara, USA), following the manufacturer's instructions. Purified products were directly sequenced using the same primers as those for PCR. DNA sequencing was carried out using BigDye® Terminator v3.1 Cycle Sequencing Kit (Applied Biosystems by Thermo Fisher Scientific, Prague, Czech Republic) and a 3130 Genetic Analyzer (Applied Biosystems). The obtained sequences were assembled and edited using Sequencher software (Gene Codes Corp., Ann Arbor, MI, USA). Newly generated sequences of 18S rDNA, 28S rDNA and COI were deposited in GenBank under accession numbers PP115569 (28S rDNA), PP115568 (18S rDNA) and PP117929-PP117934 (COI). Molecular vouchers (hologenophores, paragenophores; Pleijel *et al*., 2008) were deposited in the Institute of Parasitology, Czech Academy of Sciences, České Budějovice, Czech Republic.

**Table 2.** List of primers used for PCR amplifications of mitochondrial and nuclear markers in the present study

Locus	Primer name	Direction	Sequence (5́–3́)	Size of the fragment (bp)	Ta (°C)	References
18S	18SF	Forward	AAG GTG TGM CCT ATC AAC T	1383	52°C	Song *et al*. (2008)
	18SR	Reverse	TTA CTT CCT CTA AAC GCT C			
28S	28SF	Forward	ACA ACT GTG ATG CCC TTA G	668	52°C	Song *et al*. (2008)
	28SR	Reverse	TGG TCC GTG TTT CAA GAC G			
CO1	LCO1490	Forward	GGT CAA CAA ATC ATA AAG ATA TTG G	675	45°C	Folmer *et al*. (1994)
	ErgHCO	Reverse	TAR ACY TCM GGR TGA CCR AAA AAY CA			Present study

Ta, annealing temperature.

To investigate the phylogenetic position of *Dermoergasilus madagascarensis* n. sp., to the representatives of parasitic Cyclopoida, the sequences of 28S rDNA of the species belonging to 9 genera were retrieved from GenBank and Bold databases (for details, see Table 3). Three species of the family Lernaeidae, *Lernaea cyprinacea* (Linnaeus, 1758), *Lamproglena chinensis* Yü, 1937 and *Lamproglena orientalis* Markevich, 1936 were used as outgroup. Sequences were aligned using MAFFT v.7 (Katoh and Standley, 2013). Gaps and ambiguously aligned regions were removed from the alignments with Gblocks v0.91b (Talavera and Castresana, 2007) using settings for a less stringent selection. ModelFinder (Kalyaanamoorthy *et al*., 2017) was employed to select the most appropriate model of DNA evolution. The most suitable evolutionary model for the partial sequence of 28S rDNA was TIM3 + F + I. The phylogenetic reconstruction was performed using maximum likelihood (ML) and Bayesian inference (BI) methods. ML analyses were run using IQ-TREE (Nguyen *et al*., 2015) on the W-IQ-TREE webserver (Trifinopoulos *et al*., 2016) and nodal support for the tree was assessed through ultrafast bootstrap approximation with 1000 replicates (Hoang *et al*., 2018). BI analysis was carried out in MrBayes 3.2.6 (Huelsenbeck and Ronquist, 2001) using the CIPRES platform (Miller *et al*., 2010), the analysis included 2 simultaneous runs of Markov chain Monte Carlo for 10^6^ generations, sampling every 100 generations, with a ‘burn-in’ of 25%. The results were checked in Tracer v. 1.7.1 (Rambaut *et al*., 2018) to assess chain convergence. The trees were visualized and edited in FigTree v. 1.4.3 (Rambaut, 2012). Genetic distances (uncorrected p-distance) were calculated in MEGA v. 11 (Tamura *et al*., 2021).

**Table 3.** List of parasitic copepods used for phylogenetic analyses and calculation of p-distances, including their host species, collection locality, and accession numbers for partial 18S, 28S rDNA and COI sequences from database GenBank and Bold (indicated with *)

Parasite species	Host species	Host family	Locality	GenBank/Bold accession numbers		COI	References
				18S	28S		
**Ergasilidae**							
*Acusicola margulisae*	*Amphilophus citrinellus; Oreochromis* sp.	Cichlidae	Nicaragua	MN852694	MN852851	MN85438–MN85470	Santacruz *et al*. (2020)
***Dermoergasilus madagascarensis* n. sp.**	***Paretroplus polyactis***	**Cichlidae**	**Canal des Pangalanes, Madagascar**	**PP115568**	**PP115569**	**PP117929–PP117934**	**Present study**
*Ergasilus anchoratus*	*Tachysurus fulvidraco*	Bagridae	Baoan Lake, China	DQ107564	DQ107528	–	Song *et al*. (2008)
*Ergasilus auritus*	*Gasterosteus aculeatus*	Gasterosteidae	Nova Scotia, Canada	–	–	ECTCR091*	–
*Ergasilus briani*	*Misgurnus anguillicaudatus*	Cobitidae	Dangjiangkou, China	DQ107572	DQ107532	–	Song *et al*. (2008)
*Ergasilus caeruleus*	*Lepomis gibbosus x macrochirus; L. gibbosus; L. macrochirus; Notropis* sp.; plankton	Centrarchidae, Cichlidae	Lake Opinicon, Canada; Ottawa River, Canada; Oneida Lake, USA	–	–	ECTCR003*, ECTCR005*, ECTCR006*, ECTCR007*, ECTCR008*, ZOOPS258*, ZOOPS259*, ZOOPS260*, ZOOPS351*, ZOOPS353*, ZOOPS432*, ZOOPS433*	
*Ergasilus caparti*	*Neolamprologus brichardi*	Cichlidae	Lake Tanganyika, Burundi	OQ407468	OQ407472	–	Míč *et al*. (2023)
*Ergasilus centrarchidarum*	*Ambloplites rupestris; Lepomis gibbosus; Micropterus salmoides;* plankton	Centrarchidae	Lake Opinicon, Canada; St. Lawrence River, Canada, Richelieu River, Canada; Oneida Lake, USA	–	–	ECTCR001*, ECTCR009*, ECTCR037*, ECTCR038* ECTCR052*, ECTCR053*, ECTCR054*, ECTCR055*, ZOOPS071*, ZOOPS072*, ZOOPS073*, ZOOPS074*, ZOOPS075*	–
*Ergasilus chautauquaensis*	Plankton	–	Lake Erie, USA	–	–	ZOOPS076*; ZOOPS077*; ZOOPS078*	–
*Ergasilus hypomesi*	*Acanthogobius hasta*	Gobiidae	Dangjiangkou, China	DQ107573	DQ107539	–	Song *et al*. (2008)
*Ergasilus jaraquensis*	–	–	–	–	–	MF651988, MF651989	Lima *et al*. (2017)
*Ergasilus kandti*	–	–	Kenya	–	–	–	Unpublished data
*Ergasilus lamellifer*	*Hydrocynus forskahlii*	Alestidae	Sudan	–	–	–	Unpublished data
*Ergasilus lizae*	*Fundulus diaphanus*	Fundulidae	Richelieu River, Canada	–	–	ECTCR024*, ECTCR025*, ECTCR026*, ECTCR039*	–
*Ergasilus luciopercarum*	*Perca flavescens*; plankton	Percidae	Lake Erie, Canada; Oneida Lake, USA	–	–	ECTCR078*, ECTCR079*, ECTCR080*, ZOOPS060*, ZOOPS061* ZOOPS062*, ZOOPS063*, ZOOPS064*, ZOOPS628*, ZOOPS629*, ZOOPS630*	–
*Ergasilus macrodactylus*	*Gnathochromis permaxillaris*	Cichlidae	Lake Tanganyika, Burundi	OQ407465	OQ407470	–	Míč *et al*. (2023)
*Ergasilus megacheir*	*Simochromis diagramma*	Cichlidae	Lake Tanganyika, Burundi	OQ407466	OQ407471	–	Míč *et al*. (2023)
*Ergasilus megaceros*	Plankton	–	Dickinson Lake, USA; Oneida Lake, USA	–	–	ZOOPS065*, ZOOPS066*, ZOOPS067*, ZOOPS068*, ZOOPS069*, ZOOPS070*, ZOOPS257*, ZOOPS437*, ZOOPS438* ZOOPS440*, ZOOPS439*, ZOOPS441*	–
*Ergasilus nodosus*	Bagrus bajad	Bagridae	Sudan	–	–	–	Unpublished data
*Ergasilus parasarsi*	*Simochromis diagramma*	Cichlidae	Lake Tanganyika, Burundi	OQ407467	OQ407473	–	Míč *et al*. (2023)
*Ergasilus parasiluri*	*Tachysurus fulvidraco*	Bagridae	Dangjiangkou, China	DQ107568	DQ107536	–	Song *et al*. (2008)
*Ergasilus parvitergum*	–	–	Kerala Coast, India	–	–	OP871074	Reshmi and Kappalli (2022)
*Ergasilus parvus*	*Spathodus erythrodon*	Cichlidae	Lake Tanganyika, Burundi	OQ407469	OQ407474	–	Míč *et al*. (2023)
*Ergasilus peregrinus*	*Siniperca chuatsi*	Sinipercidae	Dangjiangkou, China	DQ107577	DQ107531	–	Song *et al*. (2008)
*Ergasilus scalaris*	*Tachysurus dumerili*	Bagridae	Poyang Lake, China	DQ107565	DQ107538	–	Song *et al*. (2008)
*Ergasilus sieboldi*	*Perca fluviatilis*, *Sparus aurata*	Percidae, Spaidae	U Jezu, Czech Republic	MW810238	MW810242	–	Kvach *et al*. (2021)
*Ergasilus* sp. 1	*Clarias gariepinus*	Clariidae	Sudan	–	–	–	Unpublished data
*Ergasilus* sp. 2	–	–	Kenya	–	–	–	Unpublished data
*Ergasilus tumidus*	*Acheilognathus taenianalis*	Acheilognathidae	Niushan Lake, China	DQ107569	DQ107535	–	Song *et al*. (2008)
*Ergasilus versicolor*	–	–	Oneida Lake, USA	–	–	ZOOPS261*, ZOOPS262*, ZOOPS263*, ZOOPS264*, ZOOPS265*	–
*Ergasilus wilsoni*	–	–	South Korea	KR048765	KR048843	KR049036	Baek *et al*. (2016)
*Ergasilus yaluzangbus*	*Oxygymnocypris stewartii*	Cyprinidae	Lasa River, Tibet	DQ107578	DQ107540	–	Song *et al*. (2008)
*Gamispinus diabolicus*	–	–	–	MF651978	–	MF651982, MF651983	Lima *et al.* (2017)
*Miracetyma* sp.	–	–	–	MF651981	–	MF651984, MF651985, MF651986, MF651987	Lima *et al*. (2017)
*Neoergasilus japonicus*	*Lepomis gibbosus*, *Scardinius erythrophthalmus*	Centrarchidae, Cyprinidae	Rohlík, Czech Republic; U Jezu, Czech republic; Hvězda, Czech republic; Babice, Czech republic; South Korea	MH167970	MH167968	KR049037, MZ964932, MZ964933, MZ964934, MZ964935, MZ964936	Ondračková *et al*. (2019); Kvach *et al*. (2021) and Vasquez *et al*. (2022)
*Paeonodes subviridis*	–	–	Kerala Coast, India	–	–	OP425700	Reshmi and Kappalli (2022)
*Paraergasilus brevidigitus*	*Cyprinus carpio*	Cyprinidae	Tangxun Lake, China	DQ107576	DQ107530	–	Song *et al*. (2008)
*Paraergasilus longidigitus*	*Abramis brama, Perca fluviatilis, Scardinius erythrophthalmus*	Leuciscinae, Percidae,	Pahrbek, U Jezu, Czech Republic	MW810239	MW810243	–	Kvach *et al*. (2021)
*Paraergasilus medius*	*Ctenopharyngodon idella*	Xenocyprididae	Tangxun Lake, China	DQ107574	DQ107529	–	Song *et al*. (2008)
*Sinergasilus major*	*Ctenopharyngodon idella*	Xenocyprididae	Tangxun Lake, China; Danube River	DQ107558	DQ107524	–	Song *et al*. (2008)
*Sinergasilus polycolpus*	*Hypophthalmichthys molitrix*	Xenocyprididae	Tangxun Lake, China; Jingzhou, China	DQ107563	DQ107525	KR263117	Song *et al*. (2008) and Feng *et al*. (2016)
*Sinergasilus undulatus*	*Cyprinus carpio*	Cyprinidae	Tangxun Lake, China	DQ107563	DQ107525	MW080644	Song *et al*. (2008) and Hua (2020)
*Therodamas longicollum*	*Leporinus fasciatus*	Anostomidae	Jarilandia, Brazil	MW652731	–	–	Oliveira *et al*. (2021)
**Lernaeidae**							
*Lamproglena chinensis*	*Channa argus*	Channidae	Dangjiangkou, China	DQ107553	DQ107545	–	Song *et al*. (2008)
*Lamproglena orientalis*	*Chanodichthys dabryi*	Xenocyprididae	Tangxun Lake, China	DQ107549	DQ107542	–	Song *et al*. (2008)
*Lernaea cyprinacea*	*Chanodichthys erythropterus*	Xenocyprididae	Dongxi Lake, China; Jingzhou, China	DQ107555	DQ107547	KM235194	Song *et al*. (2008) and Su *et al*. (2016)

Newly generated sequence is given in bold.

## Results

Endemic cichlid *P. polyactis* from the Canal des Pangalanes (locality 4 in Fig. 1) was the only host species (out of 15 species examined) infected by parasitic copepods and exhibited intensity of infection ranging from 5 to 283 (mean 59) per individual fish. Overall, 20 specimens of *P. polyactis* were examined and the prevalence of *Dermoergasilus* parasites was 90%. Total prevalence of *Dermoergasilus* among all examined fishes in the study was 18%.

The copepod specimens collected from *P. polyactis* were identified as *Dermoergasilus* based on the diagnostic morphological characters according to Ho and Do (1982), specifically: (i) antenna, except terminal claw, covered with inflated transparent membrane; (ii) paired caudal rami each with a digitiform process; and (iii) middle segment of endopod of legs II and III possessing a single seta.

**Family Ergasilidae Burmeister, 1835**

**Genus *Dermoergasilus* Ho & Do, 1982**

*Dermoergasilus madagascarensis* n. sp.

***Type-host***: *Paretroplus polyactis* (Bleeker, 1878) (Cichlidae, Cichliformes)

***Type-locality***: Canal des Pangalanes (at Andevoranto) (18°57′17.50″S, 49° 6′29.90″E), Madagascar

***Type and voucher material***: Holotype (adult female): Cr-39 (1 specimen). Paratypes (adult females): Cr-39 (3 specimens). Hologenophores (adult females): Cr-39 (16 specimens).

***Site on host***: Gill filaments.

***Prevalence and intensity of infection****:* 90% (18 fish infected/20 fish examined); 5–283 (mean 59) copepods per infected host.

***ZooBank registration***: urn:lsid:zoobank.org:act:5A5C2DCB-CCAB-4545-B6F4-F416CC22B10D

***Representative DNA sequences***: A 1384 bp long 18S rDNA sequence, 674 bp long 28S rDNA sequence and 9 COI sequences of 678 bp long obtained from 10 specimens are deposited in the NCBI GenBank database under the accession numbers PP115569 (28S rDNA), PP115568 (18S rDNA) and PP117929-PP117934 (COI), respectively.

***Etymology***: The species was named after the type locality, Madagascar Island, from which it was first discovered.

### Description

**Figure 2.**
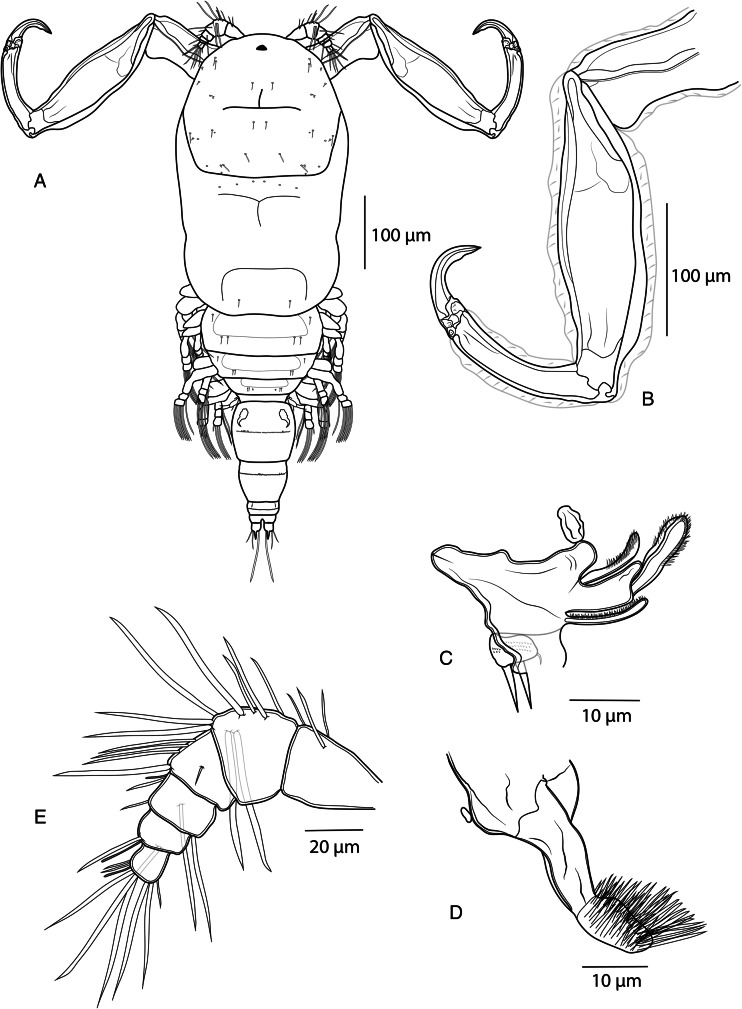
*Dermoergasilus madagascarensis* n. sp., adult female from *Paretroplus polyactis*. (A) habitus, dorsal; (B) antenna, ventral; (C) mandible and maxilulle, ventral (D) maxilla, ventral; (E) antennule, ventral.

**Figure 3.**
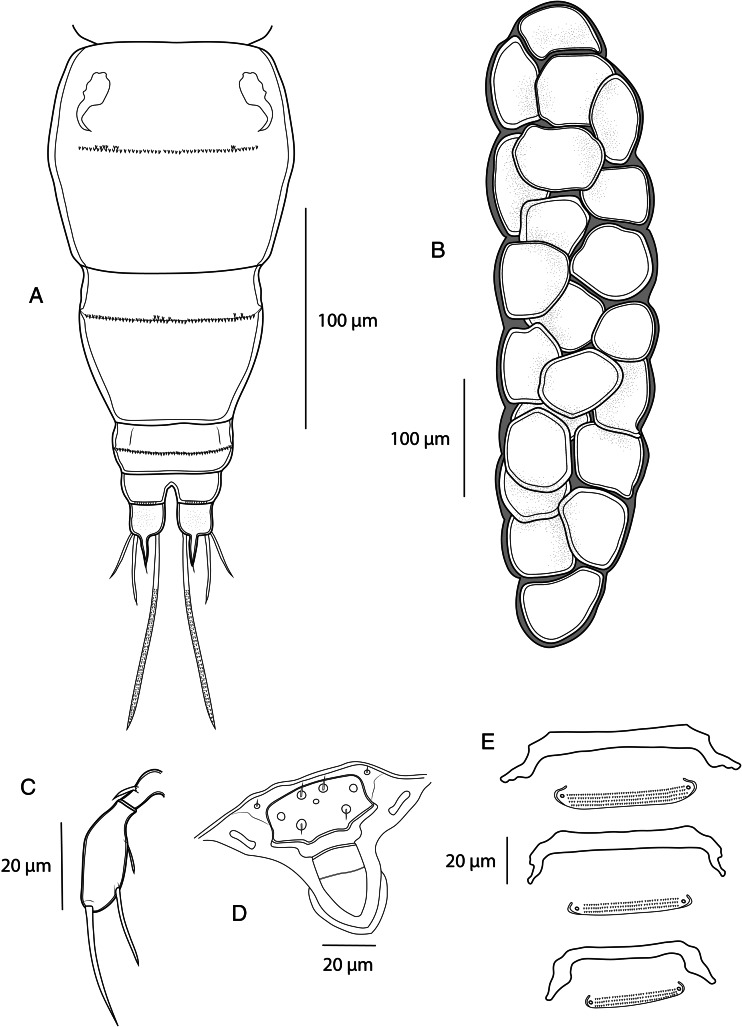
*Dermoergasilus madagascarensis* n. sp., adult female from *Paretroplus polyactis*. (A) abdomen and caudal rami; (B) egg sac, dorsal; (C) leg 5, ventral; (D) rostrum, dorsal; (E) interpodal plates, ventral.

**Figure 4.**
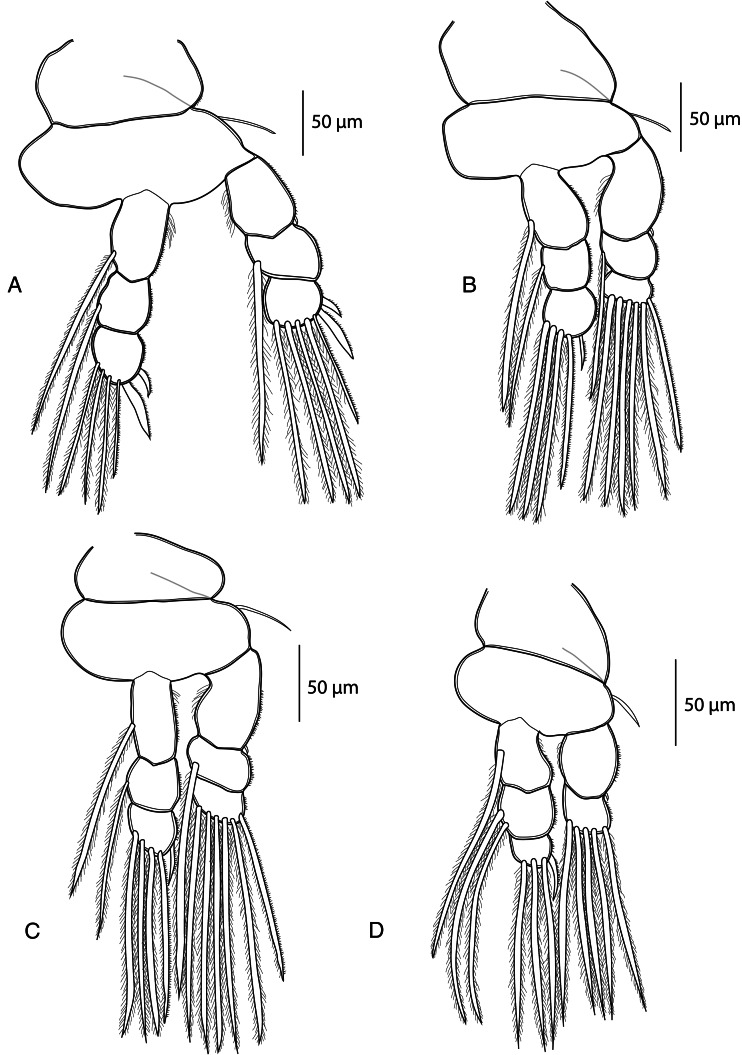
*Dermoergasilus madagascarensis* n. sp., adult female from *Paretroplus polyactis*. (A) leg 1, ventral; (B) leg 2, ventral; (C) leg 3, ventral; (D) leg 4, ventral.

**Figure 5.**
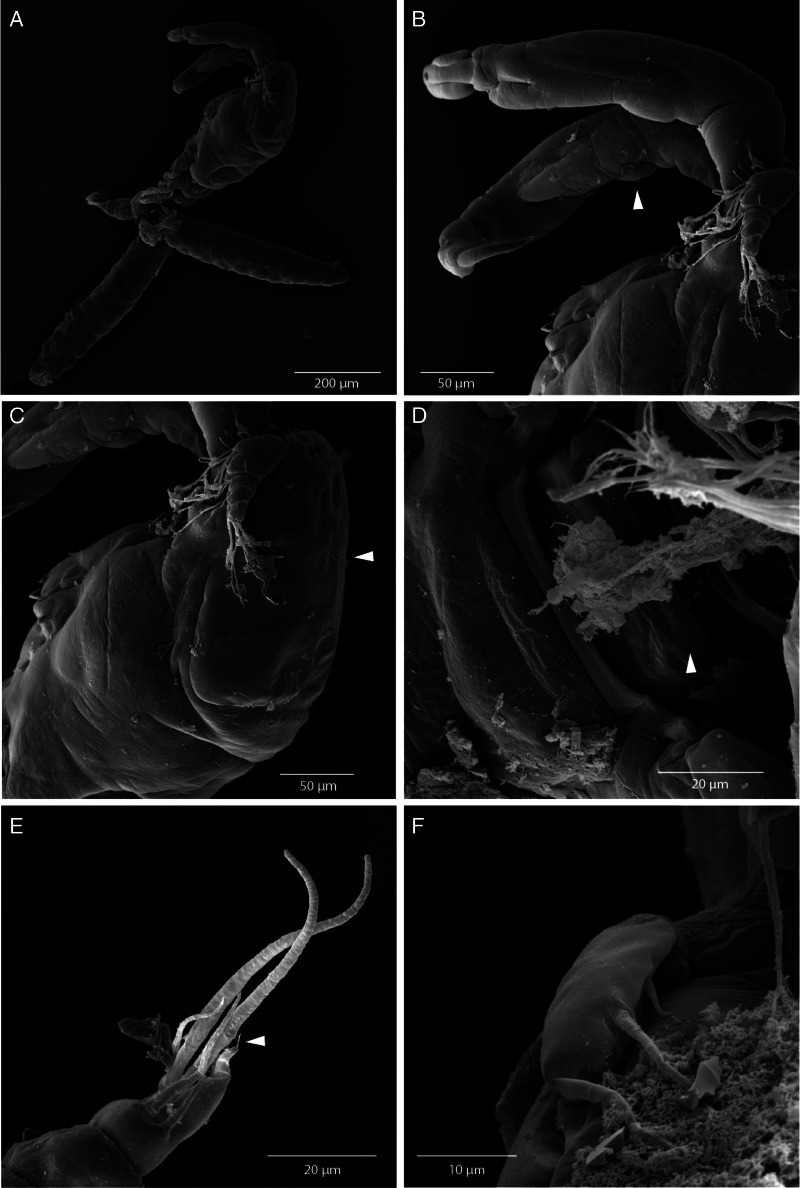
Scanning electron micrographs of *Dermoergasilus madagascarensis* n. sp., adult female from *Paretroplus polyactis*. (A) entire female body, carrying egg sacs, lateral; (B) antenna with transparent membrane (arrow), dorsal; (C) cephalosome with sensory setae and pits (arrow); antennule, lateral dorsal; (D) interpodal plates with ornamentation (arrow), ventral; (E) caudal rami and digitiform process (arrow), ventral; (F) leg 5, ventral.

*Adult female.* [Based on 10 specimens; Figs 2–5; measurements in Table 4].

**Table 4.** Measurements (in micrometers) of specimens (*n* = 10) of *Dermoergasilus madagascarensis* n. sp. parasitizing endemic cichlid *Paretroplus polyactis* in Madagascar

Character	Range	Mean
Total length	610–754	695
Body width	207–239	223
Cephalosome length	207–253	226
Cephalosome width	210–266	234
Antennule length	105–118	110
Antenna length	474–509	485
Antennal segment 1 length	105–135	117
Antennal segment 2 length	180–215	198
Antennal segment 3 length	106–117	112
Antennal segment 4 (claw) length	52–66	57
Cephalothorax length	352–371	364
Cephalothorax width	238–294	260
Thoracic segment 2 length	51–61	57
Thoracic segment 2 width	145–167	157
Thoracic segment 3 length	38–48	43
Thoracic segment 3 width	104–118	112
Thoracic segment 4 length	30–34	32
Thoracic segment 4 width	75–79	77
Thoracic segment 5 length	13–18	17
Thoracic segment 5 width	58–77	67
Genital double somite length	79–95	88
Genital double somite width	84–100	92
Abdominal segment 1 length	42–62	50
Abdominal segment 1 width	56–73	66
Abdominal segment 2 length	18–23	20
Abdominal segment 2 width	45–55	51
Abdominal segment 3 length	15–18	16
Abdominal segment 3 width	36–46	43
Caudal ramus length	14–16	15
Caudal ramus width	15–20	17
Egg-sac length	420–589	519
Egg-sac width	112–125	118

Prosome 5-segmented, composed of cephalothorax and 3 free pedigerous somites (PS-1 to PS-4) (Fig. 2A). Cephalosome roughly pentagonal, rounded and slightly tapering anteriorly; antennules and antennae visible in dorsal view (Fig. 5A and B). Cephalic ornamentation comprising inverted T-shaped marking, sensory setae and pits with bilaterally symmetrical distribution on dorsal side. Rostrum shieldlike with 6 sensillae and 3 integumental pores (Figs. 3D and 5C). PS-1 elongated, with bilateral indentations just posterior to midlength; dorsal surface with slight T-shaped and rectangular depression situated anterior and posterior, respectively, to the constricted part; dorsal ornamentation comprising circular indentations situated just posterior to cephalosome and pair of sensillae near posterior margin. PS-2 to PS-4 decreasing gradually in width posteriorly, the three together barrel-shaped. Dorsal surface of each segment possessing anteriorly arising trapezoidal plate, sensillae and pits with bilaterally symmetrical distribution.

Urosome comprising fifth pedigerous somite (PS-5), genital double somite, and 3 free abdominal somites (AS-1 to AS-3) (Fig. 3A). PS-5 reduced, smaller and thinner than prosome somites, unornamented. Genital segment large, barrel-shaped, with transverse row of spinules and pair of hook-shaped ornamentation on ventral side. Free abdominal somites decreasing in width posteriorly. AS-1 wider than long (1.2–1.3 times), almost 3 times larger than AS-2, bearing transverse row of spinules at widest part. AS-2 slightly larger than AS-3, with transverse row of spinules at midlength. AS-3 (anal somite) deeply incised posteromedially, with spinules on posterior margin.

Caudal rami nearly equal in length with AS-3, slightly wider than long; each projecting into tapering digitiform process (about 1.6 times longer than body of ramus) with inconspicuous terminal seta (Fig. 5E) and bearing 3 terminal setae – the innermost longest and thickest, ornamented with transversal rings of inconspicuous scales at posterior 3/4; 2 lateral setae longer than digitiform processes. Two cylindrical egg-sacs, much longer than wide (4 times), each composed of 2–4 rows of eggs (Fig. 3B).

Antennule (Figs 2E and 5C) 6-segmented, tapering, distally armed with simple setae; setal formula from proximal to distal segments: 3–9 – 5–4 + ae – 2 + ae – 7 + ae. Antenna (Figs 2B and 5B) comprising coxobasis, 3-segmented endopod (Enp-1 to Enp-3), and strongly recurved terminal claw. Enp-1 (proximal) longest, nearly 1.7 times longer than coxobasis, slightly inflated medially, unornamented; Enp-2 (medial) elongated, slightly curved, about half length of Enp-1, unornamented; ES-3 inconspicuous, unornamented. Terminal claw curved, about half size of ES-2, with inconspicuous subterminal inner denticle. Antenna (except terminal claw) covered with inflated cuticular membrane, without setules, spines or indentations.

Mouthparts (Fig 2C and D) comprising mandible, maxillule and maxilla; maxilliped absent. Mandible consisting of 3 blades (anterior, middle and posterior); anterior blade with sharp teeth on anterior margin; middle blade with sharp teeth on both margins; and posterior blade with sharp teeth on anterior margin. Maxillule a single lobe, ornamented with rows of tiny spinules, bearing 2 equally long outer setae and minute inner seta. Maxilla 2-segmented, comprising syncoxa and basis; syncoxa small, unarmed; basis elongated, medially slightly curved, distally with numerous sharp teeth on anterior side.

Swimming legs (L1–L4) biramous; each comprising coxa, basis, endopod (inner ramus), and exopod (outer ramus) (Fig. 4). Intercoxal sclerites slender; each with tapering ends directed posterolaterally, unornamented. Interpodal plates slender, uniform in shape; each with 2 inconspicuous bilateral pores and 3–4 transversal rows of spinules (Figs 3E and 5D). Armature formula of L1–L4 (spines – Roman numerals; setae – Arabic numerals) shown in Table 5.

**Table 5.** Spine (Roman numerals) and setal (Arabic numerals) formula of swimming legs of *D. madagascarensis* n. sp.

	Coxa	Basis	Exopod	Endopod
Leg 1	0–0	1–0	I-0; 0–1; II-5	0–1; 0–1; II-4
Leg 2	0–0	1–0	I-0; 0–1; 0–6	0–1; 0–1; I-4
Leg 3	0–0	1–0	I-0; 0–1; 0–6	0–1; 0–1; I-4
Leg 4	0–0	1–0	I-0; 0–5	0–1; 0–2; I-3

Coxa of all legs unarmed; coxa of L1 with a row of spinules extending along its outer posterior margin. Basis of all legs armed with proximal outer spine, unornamented. Legs 1–4 with outer margin of both rami ornamented with rows of spinules; outer and inner margin of first endopodal and exopodal segment, respectively, of all legs partly or completely covered with bristles.

Leg 1 (Fig. 4A): exopod 3-segmented; first segment with small naked spine arising from outer posterior margin; second segment with inner plumose seta; third segment with 2 blade-like serrated spines (shorter more proximal), 1 semi-plumose seta (=seta with outer margin serrated) and 4 plumose setae.

Endopod 3-segmented; first and second segment each with 1 plumose seta; third segment with 3 plumose setae, 1 semi-plumose seta and 2 blade-like serrated spines.

Leg 2 (Fig. 4B): exopod 3-segmented; first segment with small outer spine; second segment with 1 plumose seta; third segment with 1 semi-plumose seta and 5 plumose setae.

Endopod 3-segmented; first and second segments each with 1 small slender serrated spine, 1 plumose seta; third segment with 3 plumose setae, 1 semi-plumose seta.

Leg 3 (Fig. 4C): exopod 3-segmented; first segment with small outer spine; second segment with 1 plumose seta; third segment with 1 semi-plumose seta and 5 plumose setae. Endopod 3-segmented; first and second segments each with 1 plumose seta; third segment with 1 small slender serrated spine, 3 plumose setae and 1 semi-plumose seta.

Leg 4 (Fig. 4D): exopod 2-segmented; first segment elongated, with small outer spine; second segment with 5 plumose setae. Endopod 3-segmented; first segment with 1 plumose seta; second segment with 2 plumose setae; third segment with 1 slender serrated spine and 3 plumose setae.

Leg 5 (Figs 3C and 5F): reduced but clearly visible, 2-segmented. Basal segment very small and visible dorsally, bearing outer seta; distal segment with 3 setae on inner margin (apical seta largest).

Specimens preserved in ethanol faint brown in colour, with blue spot in eyespot and sometimes in cephalothorax.

Male: Unknown

### Remarks

*Dermoergasilus madagascarensis* n. sp. represents another species of *Dermoergasilus*, besides *D. curtus* (El-Rashidy and Boxshall, 2001) and *D. intermedius* (Kabata, 1992), that have antennae with only slightly inflated cuticular membrane. All other known *Dermoergasilus* spp. possess a conspicuous balloon-like inflated membrane covering all or only the first (in *D. semicoleus*) antennal endopodal segment. In *D. curtus*, however, the cuticular membrane covers only the inner surface of the first endopodal segment of the antenna, whereas in *D. intermedius* and *D. madagascarensis* n. sp. the membrane ensheathes all endopodal segments. The new species differs further from *D. curtus* mainly by having: (i) a pentagon-shaped cephalosome (*vs* bullet-shaped cephalosome); (ii) second endopodal segment of the antenna without a minute seta (*vs* with a minute seta proximally on inner margin of the segment); (iii) interpodal plates ornamented with 3–4 rows of spinules (*vs* 1 row of spinules); (iv) genital segment with 1 medial row of spinules (*vs* 3 posterior rows of spinules); (v) urosomites without folded membrane; and (vi) 2 lateral caudal setae longer than the digitiform process (*vs* shorter than the digitiform process). *Dermoergasilus madagascarensis* n. sp. is easily differentiated from *D. intermedius* by having: (i) anteriorly rounded and slightly tapering pentagonal cephalosome (*vs* anteriorly flat square-shaped cephalosome with widely separated antennules); (ii) second endopodal segment of the antenna medially swollen (*vs* the segment slender and of the same diameter along entire antenna); (iii) interpodal plates ornamented with 3–4 rows of spinules (*vs* unornamented); (iv) genital segment with 1 medial row of spinules (*vs* 1 posterior row of spinules, sometimes with gaps in middle part); (v) 2 lateral caudal setae longer than the digitiform processes (*vs* 1 longer and 1 shorter than the digitiform process); and (vi) a different armature formula of the third endpodal segment of legs II to IV.

In terms of the armature of the swimming legs, *D. madagascarensis* n. sp. shares the same spine and setal formula with 6 other species of *Dermoergasilus*, namely *D. amplectens*, *D. cichlidus, D. curtus, D. longiabdominalis, D. occidentalis* and *D. semiamplectens*, recorded on fishes of different families, but mostly of the Mugilidae (see Table 1). With the exception of *D. curtus*, all 5 species mentioned above are clearly distinguished from the new species by having a slender urosomite (genital segment and the first abdominal somite are markedly elongated *vs* both barrel shaped in *D. madagascarensis* n. sp.).

*Dermoergasilus madagascarensis* n. sp. is the first recorded copepod parasitizing freshwater fishes in Madagascar and besides *D. amplectens* from orange chromid *Pseudetroplus maculatus* (Bloch) (India; Ho *et al*., 1992) and *D. cichlidus* from *Coptodon zilii* (Iraq; Ali and Adday, 2019), it is the third species of *Dermoergasilus* hitherto recorded from the gills of a cichlid fish.

*Molecular characterization and phylogenetic position of* D. madagascarensis *n. sp. within the Ergasilidae*

Partial fragments of 18S (1384 bp), 28S (674 bp) rDNA and COI (675 bp) were obtained from 10 individuals of *D. madagascarensis* n. sp. No intraspecific sequence variability was found for any of nuclear ribosomal markers (partial 18S and 28S rDNA). Six haplotypes were found in the COI mtDNA with a low intraspecific genetic variation of 0.15–1.48%. Genetic comparison of *D. madagascarensis* n. sp. with other Ergasilidae species showed the lowest interspecific genetic distance with *Ergasilus megaceros* Wilson, 1916 (17.7%) and highest interspecific genetic distance with *Neoergasilus japonicus* (Harada, 1930) (23.9%) for COI sequences (Table 6). When comparing *D. madagascarensis* n. sp. to other ergasilid species in rDNA sequence data, the minimum interspecific distances were observed with *E. sieboldi* von Nordmann, 1832 (0.9% for 18S rDNA, and 4.3% for 28S rDNA) and maximum interspecific divergences were observed with *Therodamas longicollum* Oliveira *et al*. (2021) (3.4% for 18S rDNA) and *Sinergasilus major* (Markevich, 1940) (10.9% for 28S rDNA).

**Table 6.** Interspecific genetic variabilities of family Ergasilidae

		1	2	3	4	5	6	7	8	9	10
**1**	***Dermoergasilus*** (**1, 1, 1)**		**21.3–22.4**	**17.7–23.6**	**20.8–23.9**	–	**19.5–22.1**	**20.6–21.2**	**21.4–22.1**	**22.4–23.0**	–
2	*Acusicola* (1, 1, 1)	**2.1**		16.7–22.9	17.4–18.4	–	18.4–20.4	20.8–21.8	20.6–21.6	20.9–21.6	–
		**5.8**									
3	*Ergasilus* (13, 14, 11)	**0.8–2.7**	1.6–3.0		18.1–21.9	–	16.4–22.0	17.1–21.2	20.5–23.8	19.5–24.8	–
		**4.3–9.6**	5.3–9.9								
4	*Neoergasilus* (1, 1, 1)	**1.6**	1.9	0.5–2.0		–	17.3–20.1	20.1–21.2	19.9–20.6	20.9–21.5	–
		**7.5**	8.9	5.6–11.2							
5	*Paraergasilus* (3, 3, 0)	**1.6–1.9**	2.3–2.4	1.2–3.0	2.1–2.3		–	–	–	–	–
		**5.4–6.1**	5.5–6.0	3.5–9.4	7.2–7.5						
6	*Sinergasilus* (3, 3, 2)	**1.7–2.1**	1.7–1.9	0.5–2.8	1.6–1.7	2.1–2.4		18.8–19.5	19.1–21.6	20.6–21.4	–
		**8.8–10.9**	9.6–11.4	8.0–12.6	11.0–12.6	8.6–10.9					
7	*Gammispinus* (1, 0, 1)	**1.5**	1.9	1.4–3.3	1.5	2.0–2.4	2.0		20.8–21.4	23.1	–
		–	–	–	–	–	–				
8	*Miracetyma* (1, 0, 1)	**2.4**	2.3	1.9–4.4	3.1	2.7	3.1	2.7		26.0–26.3	–
		–	–	–	–	–	–	–			
9	*Paeonodes* (0, 0, 1)	–	–	–	–	–	–	–	–		–
		–	–	–	–	–	–	–	–		
10	*Therodamas* (1, 0, 0)	**2.9**	2.9	1.8–3.9	2.9	2.7	2.7	3.7	3.4	–	
		–	–	–	–	–	–	–	–		

Below the diagonal are showed the values for 18S rDNA (first line) and 28S rDNA(second line) and above the diagonal for COI. The range indicates minimum and maximum value of the genetic variability for species of the genus. Numbers in brackets indicate the number of species with available sequences for the specific marker (18S, 28S, COI). Bold numbers only indicate values for the genus *Dermoergasilus*, which is the focus of this article.

**Figure 6.**
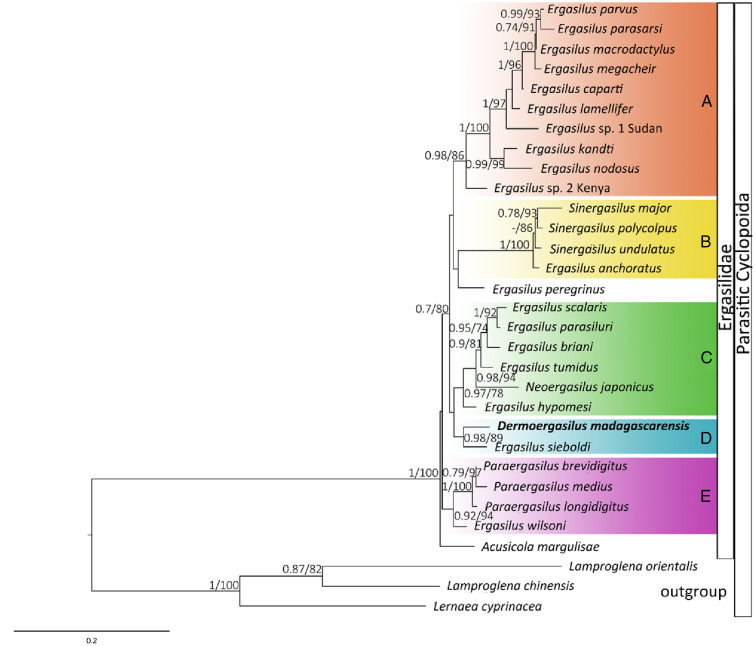
Phylogenetic tree of Ergasilidae reconstructed by Maximum Likelihood. The tree is based on the partial 28S rDNA sequences (674 bp alignment). Values along the branches indicate posterior probabilities from Bayesian Inference and bootstrap values from Maximum Likelihood (dashes indicate values below 0.7 and 50, respectively).

ML and BI analyses based on 28S rDNA sequences of Ergasilidae yielded trees with congruent topologies with similar nodal support values and revealed 5 well-supported groups (Fig. 6): (A) African *Ergasilus* species group; (B) Asian *Sinergasilus* species and the *Ergasilus anchoratus* Markevich, 1946 group; (C) Asian *Ergasilus* species and the *Neoergasilus japonicus* group, (D) *E. sieboldi* and *D. madagascarensis* n. sp. group and (E) *Paraergasilus* species and the *Ergasilus wilsoni* Markevich, 1933 group. Phylogenetic reconstruction showed the polyphyletic status of the genus *Ergasilus*.

## Discussion

Diversity of fish ectoparasites in native Malagasy freshwater fish has been little studied in the past. The present study was a part of large parasitological investigation performed only in 4 localities of north-western Madagascar, however, documenting unknown diversity of fish parasites in isolated freshwater region with endemic fish fauna (i.e., Madagascar), the pattern which was previously shown for endemic freshwater fish in other regions i.e., Peri-Mediterranean and Middle East (Benovics *et al*., 2017, 2021; Rahmouni *et al*., 2017; Řehulková *et al.*, 2020; Nejat *et al*., 2023). Prior to this study, 12 valid species of *Dermoergasilus* were known, including 1 species, specifically *D. longiabdominalis*, in mugilid hosts in Madagascar. Two *Dermoergasilus* species were previously reported on cichlid hosts in India and Iraq. The first species, *D. amplectens*, was recorded on a number of fish species and over a wide geographic range, including *Pseudetroplus maculatus*, an endemic cichlid of southern India and Sri Lanka. The second species, *D. cichlidus*, was described from *Coptodon zillii*, a non-native cichlid in Iraq. *Dermoergasilus madagascarensis* n. sp. represents the third species of the genus reported on cichlids and the second species of the genus revealed in Madagascar and a single known species currently known only from endemic Malagasy cichlids (i.e., *P. polyactis*).

Even though questioned in the past (Gussev, 1987; Kabata, 1992; El-Rashidy and Boxshall, 2001), *Dermoergasilus* still remains valid. From the 3 morphological characters proposed by Ho and Do, 1982 only 1 clearly differentiates this genus, which is a digitiform process on each paired caudal rami. The other 2 characters seem to be ambiguous. The inflated transparent membrane is quite a vague morphological character, and some species of *Dermoergasilus* do not have it well developed (e.g. *D. curtus* or *D. intermedius*). The membrane could be an ancestral trait that is being lost during the evolution, from clearly visible balloon-like inflation in *D. amplectens* to barely noticeable cuticle in *D. curtus*. Moreover, there are some *Ergasilus* species with some kind of hyaline membrane on antenna. For example, the membrane on antenna of *Ergasilus megacheir* (Sars, 1909) appears to be very similar to that of *D. curtus*. The middle segment of endopod of legs II and III possessing a single seta is even less persuasive character, since at least 10 *Ergasilus* species (e.g. *E. tumidus* Markevich, 1940, *E. briani* Markevich, 1933, *E. gibbus* von Nordmann, 1832, *E. gobiorum* Markevich & Sukhnenko, 1967 etc.) also possess this character (Ho *et al*., 1992; Kabata, 1992). There are other morphological traits present in most of the species of *Dermoergasilus*, e.g. long first free abdominal segment, similar morphology of leg 5, falciform seta on legs, some species even share the same spine-seta and antennal formula. However, neither of them can clearly distinguish *Dermoergasilus* from other members of Ergasilidae but could indicate their possible close relationship and a common ancestry.

Based on the literature review, *D. madagascarensis* n. sp. shares the same spine and setal formula with 6 other species of the genus. Future studies using molecular analyses should focus on this aspect and verify, if species with the same armature of swimming legs are phylogenetically related. Many of these species were recorded from mugilid hosts in Indian region. It is possible that they have the common origin, and the divergence of the species is associated with geographical isolation of Madagascar, drifting away from the Indian peninsula 96–65 Mya (Vences *et al*., 2009). El-Rashidy and Boxshall (2001) suggested that a mugilid as a host is a plesiomorphic character for *Dermoegasilus*, and that the ancestor of this group of parasites also occurred on a mugilid host. Acquiring hosts of other fish families could be a result of the adaption to the conditions in the new environment, which are cichlids in this case. However, only molecular data from *D. curtus*, *D. longiabdominalis* and *D. semiamplectens* reported from mugil hosts in India, China, Madagascar, Philippines and Myanmar would shed more light on the origin of *D. madagascarensis* n. sp. and clarify its relationship with other *Dermoergasilus* and *Ergasilus* species.

Scanning electron microscopy (SEM) is the method providing the appropriated visualization of some morphological structures, in our study, specifically sensory setae and pits, and also the minute seta on the digitiform process in *D. madagascarensis* n. sp., while the latter character was not visible under the light microscope. It is highly likely that some morphological characters might be overlooked in older descriptions of Ergasilidae, in which authors did not use SEM.

The present results of phylogenetic analyses are consistent with previously reported ergasilid phylogenies (Song *et al*., 2008; Santacruz *et al*., 2020; Kvach *et al*., 2021; Míč *et al*., 2023). Phylogenetic reconstruction based on 28S rDNA presented in this study showed the sister relationship among newly described *D. madagascarensis* n. sp. and *E. sieboldi* von Nordmann, 1832, a cosmopolitan parasite of freshwater fishes (Yamaguti, 1939; Kabata, 1979; Amado *et al*., 2001). While the cephalothorax shape is similar between the 2 species, the new species differs from *E. sieboldi* by: (i) digitiform process on caudal rami (ii) absence of spines on antenna (*vs* short spine on inner surface of the first endopodal segment of antenna and 2 short spines on inner surface of the second endopodal segment of antenna in *E. sieboldi*); (iii) absence of circular structure posterior to inverted T-structure on cephalothorax (*vs* presence in *E. sieboldi*), (iv) caudal rami bearing 3 terminal setae (*vs* 4 terminal setae in *E. sieboldi*); (v) having only 1 seta on the second segment of the endopods of legs II and III (*vs* 2 setae in *E. sieboldi*).

However, we can still ask whether the position of *D. madagascarensis* n. sp. in the phylogenetic tree is because of the real relatedness of these 2 species or due to the lack of molecular data for other species of the family Ergasilidae, especially those currently included in *Dermoergasilus*. A close relationship among *D. madagascarensis* n. sp. and African species of *Ergasilus* has not been confirmed in present study, so the newly described species does not appear to originate from Africa (at least based on the phylogeny including currently available DNA sequences of African *Ergasilus*). A fragment of COI mtDNA gene was also successfully obtained for representative number of *D. madagascarensis* n. sp. specimens. Unfortunately, no other DNA data are currently available for representatives of the *Dermoergasilus* genus and no threshold for intra- or interspecific variability was set for ergasilid species. However, the distances between COI haplotypes of *D. madagascarensis* n. sp. did not exceed 1.5%, the intraspecific limit generally accepted for COI mtDNA of Copepoda (Bucklin *et al*., 2003; Dippenaar *et al*., 2010; Laakmann *et al*., 2013). The COI intraspecific distances in other ergasilid species reached the values from 0% (*E. wilsoni* or *E. jaraquensis* Thatcher & Robertson B.A., 1982) to 6.9% (*N. japonicus*). In contrast, COI distances between *Dermoergasilus* and other genera reached values over 17%, supporting it being a separate genus. Nevertheless, to clearly resolve the phylogeny of Ergasilidae, DNA sequences of more ergasilid species from other parts of the world are needed.

## Conclusion

Based on morphological and molecular data, a new species of *Dermoergasilus* has been described. *Dermoergasilus madagascarensis* n. sp. from the cichlid *P. polyactis* is the second report of a representative of the genus in Madagascar and the first molecular data for the genus were obtained. Even though the validity of the genus was questioned in the past, the possession of digitiform process on caudal rami clearly distinguishes it from other genera of the Ergasilidae. However, our phylogenetic analyses showed the polyphyly of the genus *Ergasilus*, and the close phylogenetic relationship between *D. madagascarensis* n. sp. and widely geographically distributed *Ergasilus sieboldi*. We highlight that more molecular data are needed to clarify the relationships between the species of *Dermoergasilus* and their position within the Ergasilidae.

## Supporting information

Míč et al. supplementary material Míč et al. supplementary material

## Data Availability

Type and voucher specimens were deposited in the Institute of Parasitology, Czech Academy of Sciences, České Budějovice, Czech Republic (accession code Cr-39). The sequences produced in this study were deposited in GenBank of NCBI at https://www.ncbi.nlm.nih.gov/ (accession codes PP115568, PP115569, PP117929-PP117934).

## Supplementary material

The supplementary material for this article can be found at https://doi.org/10.1017/S0031182024000088.
