# Supplementary material for: New species of Dermoergasilus Ho & Do, 1982 (Copepoda: Cyclopoida: Ergasilidae) parasitizing endemic cichlid Paretroplus polyactis (Bleeker) in Madagascar
Source: Parasitology. 2024 Jan 19;151(3):319–36. doi: 10.1017/S0031182024000088 (PMC11007281; doi:10.1017/S0031182024000088)
Supplement: Míč et al. supplementary material [file S0031182024000088sup001.docx]

SUPPLEMENT

**Supplementary Table 1** List of host species, localities of collection with their coordinates and fish sample size.

| **Host species** | **Locality** | **Coordinates** | **Sample size** |
| --- | --- | --- | --- |
| *Coptodon rendalli* (Boulenger) | Lake Ravelobe (1) | 16°18′23.14″S 46°48′43.32″E | 6 |
|  | crater lakes of Mont Passot (3) | 13°19′1.84″S 48°14′3.60″E | 2 |
| *Heterotis niloticus* (Cuvier) | Lake Ravelobe (1) | 16°18′23.14″S 46°48′43.32″E | 2 |
| *Glossogobius giuris* (Hamilton) | Anjingo River (2) | 14°50′40.89″S 48°14′43.36″E | 3 |
| *Glossogobius* spp. | Lake Ravelobe (1) | 16°18′23.14″S 46°48′43.32″E | 1 |
| *Oreochromis mosambicus* (Peters) | Lake Ravelobe (1) | 16°18′23.14″S 46°48′43.32″E | 3 |
|  | crater lakes of Mont Passot (3) | 13°19′1.84″S 48°14′3.60″E | 2 |
| *Oreochromis niloticus* (Linnaeus) | Lake Ravelobe (1) | 16°18′23.14″S 46°48′43.32″E | 3 |
|  | Anjingo River (2) | 14°50′40.89″S 48°14′43.36″E | 3 |
|  | Canal des Pangalanes (4) | 18°57′17.50″S 49°6′29.90″E | 5 |
| *Osteomugil robustus* (Günther) | Anjingo River (2) | 14°50′40.89″S 48°14′43.36″E | 1 |
| *Pachypanchax omalonotus* (Duméril) | Anjingo River (2) | 14°50′40.89″S 48°14′43.36″E | 2 |
| *Paratilapia polleni* Bleeker | Anjingo River (2) | 14°50′40.89″S 48°14′43.36″E | 9 |
| *Paratilapia* sp. 1 | crater lakes of Mont Passot (3) | 13°19′1.84″S 48°14′3.60″E | 1 |
| *Paratilapia* sp. 2 | crater lakes of Mont Passot (3) | 13°19′1.84″S 48°14′3.60″E | 16 |
| *Paretroplus polyactis* (Bleeker) | Canal des Pangalanes (4) | 18°57′17.50″S 49°6′29.90″E | 20 |
| *Planiliza macrolepis* (Smith) | Lake Ravelobe (1) | 16°18′23.14″S 46°48′43.32″E | 1 |
| *Ptychochromis grandidieri* (Sauvage) | Canal des Pangalanes (4) | 18°57′17.50″S 49°6′29.90″E | 5 |
| *Ptychochromis* spp. | Anjingo River (2) | 14°50′40.89″S 48°14′43.36″E | 15 |
